# Marine amoebae with cytoplasmic and perinuclear symbionts deeply branching in the *Gammaproteobacteria*

**DOI:** 10.1038/srep13381

**Published:** 2015-08-25

**Authors:** Frederik Schulz, Tomáš Tyml, Ilaria Pizzetti, Iva Dyková, Stefano Fazi, Martin Kostka, Matthias Horn

**Affiliations:** 1 Department of Microbiology and Ecosystem Sience, University of Vienna, Althanstraße 14, A-1090 Vienna, Austria; 2 Faculty of Science, University of South Bohemia, Branišovská 31, 370 05 České Budějovice, Czech Republic; 3 Institute of Parasitology, Biology Centre CAS, Branišovská 31, 370 05 České Budějovice, Czech Republic; 4 Department of Botany and Zoology, Faculty of Science, Masaryk University, 61137 Brno, Czech Republic; 5 Water Research Institute, National Research Council of Italy (IRSA-CNR), Via Salaria Km 29,300, 00015 Monterotondo - Roma, Italy

## Abstract

Amoebae play an important ecological role as predators in microbial communities. They also serve as niche for bacterial replication, harbor endosymbiotic bacteria and have contributed to the evolution of major human pathogens. Despite their high diversity, marine amoebae and their association with bacteria are poorly understood. Here we describe the isolation and characterization of two novel marine amoebae together with their bacterial endosymbionts, tentatively named ‘*Candidatus* Occultobacter vannellae’ and ‘*Candidatus* Nucleophilum amoebae’. While one amoeba strain is related to *Vannella*, a genus common in marine habitats, the other represents a novel lineage in the Amoebozoa. The endosymbionts showed only low similarity to known bacteria (85–88% 16S rRNA sequence similarity) but together with other uncultured marine bacteria form a sister clade to the *Coxiellaceae*. Using fluorescence *in situ* hybridization and transmission electron microscopy, identity and intracellular location of both symbionts were confirmed; one was replicating in host-derived vacuoles, whereas the other was located in the perinuclear space of its amoeba host. This study sheds for the first time light on a so far neglected group of protists and their bacterial symbionts. The newly isolated strains represent easily maintainable model systems and pave the way for further studies on marine associations between amoebae and bacterial symbionts.

Unicellular eukaryotes, in particular free-living amoebae, are major players in the environment. Free-living amoebae are ubiquitous in soil, fresh- and seawater, but can also be found in anthropogenic environments, such as cooling towers, water pipes and waste-water treatment plants^1^,^2^. Taxonomically, free-living amoebae are scattered across the eukaryotic tree of life, with the supergroup Amoebozoa containing a substantial part of known free-living amoebae, such as naked lobose amoebae (gymnamoebae)^3^,^4^. In total, there are more than 200 described species of gymnamoebae classified into over 50 genera^5^. A substantial proportion of this diversity is found in marine environments, and some genera represent exclusively marine lineages. Yet our current knowledge of marine amoebae is still scarce.

Free-living amoebae shape microbial communities; they control environmental food webs by preying on bacteria, algae, fungi and other protists and contribute to elemental cycles in diverse ecosystems^1^. Free-living amoebae typically take up their food by phagocytosis. However, some bacteria have developed strategies to survive digestive processes and eventually use amoebae as niche for intracellular replication^6^,^7^,^8^,^9^. Free-living amoebae are thus considered to have served as evolutionary training ground for intracellular microbes. Bacteria such as *Legionella pneumophila*, *Francisella tularensis*, or *Mycobacterium* species transiently exploit these protists as a vehicle to reach out for higher eukaryotic hosts. Others engage in long-term, stable associations with free-living amoebae^7^,^8^, which can be beneficial, neutral or parasitic for their hosts. These obligate intracellular symbionts include a diverse assemblage of phylogenetically different bacterial groups^10^,^11^,^12^ and their analysis has provided unique insights into the evolution of the intracellular life style^13^,^14^. However, virtually nothing is known about bacterial symbionts in marine amoebae.

Here we report on the isolation and characterization of two novel marine amoeba strains harboring obligate intracellular bacterial symbionts. Both bacteria represent deeply branching novel lineages in the *Gammaproteobacteria* affiliated with the *Coxiellaceae*. While one of the symbionts replicates in the amoeba cytoplasm, the other exploits a highly unusual intracellular niche, its host’s perinuclear space.

## Methods

### Amoeba isolation and cultivation

Lago di Paola is a meso-eutrophic lake located on the Tyrrhenian coast of Central Italy (Latium). Two narrow artificial channels at the northwestern and southeastern ends of the lake allow for a limited water exchange with the sea, sustaining a high degree of salinity throughout the year (33.7 during sampling). A surface water sample was collected on October 1, 2013, from station SAB2^15^. The number of protist-sized particles per milliliter lake water was determined with a Neubauer counting chamber. Between one and ten protist-sized particles were placed in wells on a 96-well plate (Corning Costar, Sigma-Aldrich, Germany) containing 200 μl artificial seawater (ASW, DSMZ 607) and *E. coli tolC*- as well as ampicillin (200 ng/ml)^11^. The amoeba strain A1, which was propagating on these plates was screened for the presence of bacterial endosymbionts with fluorescence *in situ* hybridization (FISH), and maintained in cell culture flasks (Nunclon delta-surface, Thermo Scientifc, Germany) containing ASW and *E. coli tolC*- as well as ampicillin (200 ng/ml).

Grains of wet sand collected on a sea shore (Montego Bay, Jamaica) were placed onto a MY75S agar plate and moistened daily with ASW (75%) as described previously^16^. Two weeks later, a morphologically uniform group of cells was transferred onto a new MY75S plate, and the newly established amoeba strain JAMX8 was sub-cultured either on plates or in liquid medium containing ASW and *E. coli tolC*- as well as ampicillin (200 ng/ml).

Trophozoites of both strains were observed in hanging drop preparations and documented using an Olympus BX51 microscope equipped with a Nomarski differential interference contrast (DIC) and an Olympus DP70 camera (Olympus Optical Co. Ltd, Japan). Trophozoites and cellular structures were analyzed using ImageJ software^17^.

### Transmission electron microscopy

For transmission electron microscopy (TEM), ASW in culture flasks containing strain A1 was replaced with 3% glutaraldehyde in 0.1 M Na-cacodylate buffer. Trophozoites of strain JAMX8 were fixed *in situ* on MY75S plates with the same fixative. Pelleted trophozoites were rinsed in 0.1 M Na-cacodylate buffer, post-fixed in 1% osmium tetroxide, dehydrated in graded acetone series, and embedded in Spurr’s resin. Ultrathin sections were stained with 2% uranyl acetate in 50% methanol and Reynold’s lead citrate and examined using a JEOL JEM 1010 electron microscope (Jeol Ltd, Japan) operating at 80 kV.

### Fluorescence *in situ* hybridization

Amoeba cells were harvested by centrifugation (3000 × g, 8 min), washed with ASW and left to adhere on slides for 30 min prior to fixation with 4% formaldehyde (15 min at room temperature). The samples were hybridized for two hours at 46 °C at a formamide concentration of 25% using standard hybridization and washing buffers^18^ and a combination of the following probes: symbiont specific probes JAMX8_197 (5′-GAAAGGCCAAAACCCCCC-3′) or A1_1033 (5′-GCACCTGTCTCTGCATGT-3′), together with EUK-516 (5′-ACCAGACTTGCCCTCC-3′^19^) targeting most eukaryotes and the EUB338 I-III probe mix (5′-GCTGCCTCCCGTAGGAGT-3′, 5′-GCAGCCACCCGTAGGTGT-3′, 5′-GCTGCCACCCGTAGGTGT-3′ 20) targeting most bacteria. FISH probes were designed based on a multiple 16S rRNA sequence alignment in the software ARB^21^ using the integrated probe-design tool. Furthermore, thermodynamic parameters and binding specificity were evaluated with the web-based tools mathFISH and probeCheck^22^,^23^. All probes were purchased from ThermoFisher Scientific (Germany). Cells were subsequently stained with DAPI (0.5 μg/ml in double distilled water, 3 min), washed once and embedded in Citifluor (Agar-Scientific, UK). Slides were examined using a confocal laser scanning microscope (SP8, Leica, Germany).

### DNA extraction, PCR, cloning and sequencing

DNA was extracted from infected amoeba cultures using the DNeasy Blood and Tissue Kit (Qiagen, Austria). Amoebal 18S rRNA genes were amplified by PCR using primers 18e (5′-CTGGTTGATCCTGCCAGT-3′) and RibB (5′-TGATCCTTCTGCAGGTTCACCTA-3′) at an annealing temperature of 52 °C^24^,^25^. Bacterial 16S rRNA genes were amplified using primers 616 V (5′-AGAGTTTGATYMTGGCTCAG-3′) and 1492R (5′-GGYTACCTTGTTACGACTT-3′) at an annealing temperature of 52 °C^26^,^27^. PCR reactions typically contained 100 ng template DNA, 50 pmol of each primer, 1 unit of Taq DNA polymerase (TopBio, Czech Republic for 18S rDNA; Fermentas, Germany for 16S rDNA), 10x Taq buffer with KCl and 2 μM MgCl2 and 0.2 μM of each deoxynucleotide in a total volume of 50 μl. PCR products were purified using the PCR Purification Kit (Qiagen, Germany) and cloned using the TOPO TA Cloning Kit (Invitrogen, Germany) following the manufacturer’s instructions. Nucleotide sequences were determined at Microsynth (Vienna, Austria) and Macrogen Europe (Amsterdam, Netherlands). Newly obtained rRNA gene sequences were deposited at Genbank/EMBL/DDBJ (accession numbers LC025958, LC025959, LC025974, LC025975).

### Phylogenetic analysis

To infer the phylogenetic position of the isolated amoeba strains in the Amoebozoa a representative dataset of 18S rRNA sequences from a total of 57 taxa was compiled. The length of the final trimmed alignment was 1226 nt; alternative alignments obtained by altering taxon sampling and/or trimming stringency were also analyzed to check the stability of deeper nodes. A more detailed analysis of the position of strain A1 in the Vannellidae was performed, comprising in total 19 taxa, both nominal species and unnamed sequences assigned to morphologically characterized strains. The alignment was processed as described above and trimmed to a final length of 1440 nt. Both datasets were analyzed using with RAxML 8.0.20^28^, with the GTR gamma model of evolution and rapid bootstrapping (1000 replicates). Bayesian interference analysis was computed for both datasets in MrBayes 3.1.2^29^ with default options, GTR gamma model and 10^6^ generations; burnin 25%.

For phylogenetic analysis of bacterial 16S rRNA sequences the sequence editor integrated in the software ARB was used to build alignments based on the current Silva ARB 16S rRNA database^21^,^30^, which was updated with sequences from GenBank/EMBL/DDBJ obtained by sequence homology searches using BLASTn available at the NCBI web site (National Centre for Biotechnology Information^31^,^32^). The alignment was trimmed to the length of the shortest sequence, manually curated and exported from ARB using a 50% conservation filter. The resulting alignment comprised 61 sequences and 1417 positions. For Bayesian analysis, PhyloBayes^33^ was used with two independent chains under GTR and the CAT + GTR model. Both analyses ran until convergence was reached (maxdiff < 0.1) and as burnin 25% of the sampled trees were removed. Posterior predictive tests were performed in PhyloBayes with the ppred program (sampling size 1000 trees).

## Results

### Two novel stenohaline amoebae containing bacterial symbionts

**Figure 1:**
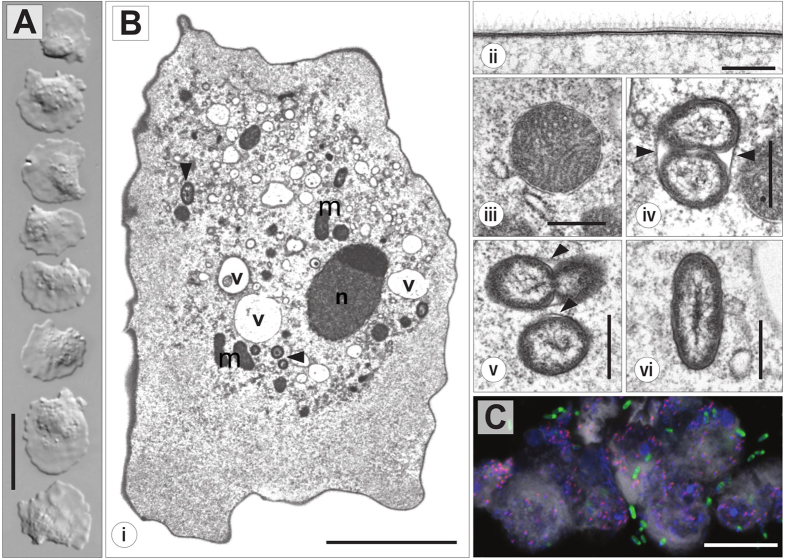
*Vannella* sp. A1 and its bacterial endosymbiont ‘*Candidatus* Occultobacter vannellae’. (**A**) Trophozoites as seen in hanging drop preparations (scale bar = 20 μm). (**B**) Fine structure of *Vannella* sp. A1 and its bacterial symbiont. (i) Section of an amoeba trophozoite: cell organelles located within granuloplasm; nucleus (n) with laterally located nucleolus, mitochondria (m), vacuoles (v), bacterial endosymbionts (arrowheads) (scale bar = 5 μm). (ii) Cell surface of trophozoite with amorphous glycocalyx (scale bar = 200 nm). (iii) Mitochondria with tubular cristae (scale bar = 500 nm). (iv–vi) Bacterial endosymbionts in detail: (iv, v) host-derived vacuolar membranes (arrowheads) enclosing endosymbionts undergoing cell division, (vi) longitudinal section through an endosymbiont (scale bar = 500 nm). (**C**) Fluorescence *in situ* hybridization image showing the intracytoplasmic location of ‘*Candidatus* Occultobacter vannellae’ (*Occultobacter*-specific probe A1_1033, pink) in its *Vannella* sp. A1 host (probe EUK516, grey) with DAPI stained nuclei (blue) and food bacteria (general bacterial probe EUB338-mix, green); scale bar indicates 10 μm.

**Figure 2:**
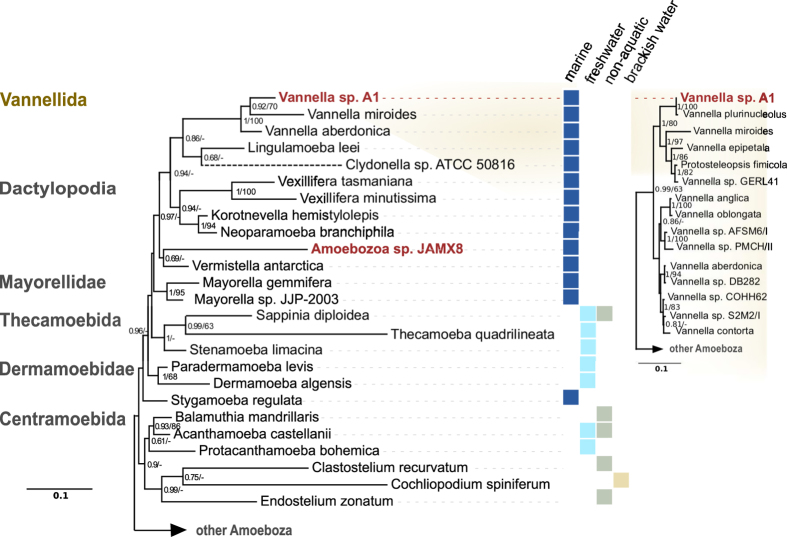
Phylogenetic relationships of *Vannella* sp. A1 and amoeba isolate JAMX8 within the Amoebozoa. Phylogenetic 18S rRNA-based trees of the Amoebozoa (left panel) and Vannellidae (right panel) constructed using the Bayesian inference method. Bayesian posterior probabilities (>0.6) and RaxML bootstrap support values (>60%) are indicated at the nodes; the dashed line indicates a branch shortened by 50% to enhance clarity. Colored squares indicate the typical habitat of the respective amoeba species (left panel). A detailed version of the trees including accession numbers is available as supplementary Figure S1.

Two novel strains of marine amoebae, initially referred to as A1 and JAMX8, were isolated from samples taken from a coastal lake in Italy and a sea shore in Jamaica, respectively. Both amoeba strains were successfully cultivated only in artificial seawater. They were truly stenohaline, shown by their incapability to grow under varying salt concentrations. In hanging drop preparations, strain A1 exhibited a flattened, oval to fan-shaped locomotive form (Fig. 1A) with an average length of 17.5 μm (S.D. 2.5, n = 50), width of 15.7 μm (S.D. 2.4, n = 50) and a length/width ratio of 0.8–1.6 (in average 1.1). The anterior hyaloplasm typically occupied about half the cell length. At the ultrastructure level, the cytoplasm contained a nucleus with a peripheral nucleolus or nucleoli, oval mitochondria and food vacuoles (Fig. 1Bi). The cell surface was covered with fine, hair-like filamented glycostyles (length of 71 ± 9 nm) (Fig. 1Bii). The mitochondria possessed branching tubular cristae (Fig. 1Biii). The partial 18S rRNA gene sequence (1889 nt) of strain A1 was most similar to *Vannella plurinucleolus* and other *Vannella* species (98% sequence similarity). In our phylogenetic analyses the placement of strain A1 in the family Vannellidae was highly supported (Figs 2 and S1) and further confirmed by a more detailed analysis focusing on the Vannellidae only, in which *V. plurinucleus* appeared as closest relative (Fig. 2 inset, S1).

**Figure 3:**
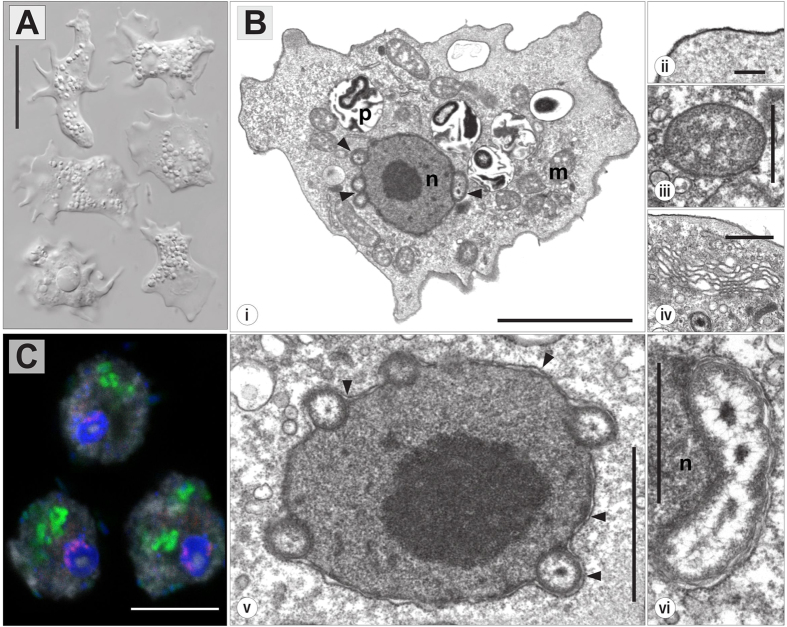
Amoeba isolate JAMX8 and its bacterial endosymbiont ‘*Candidatus* Nucleophilum amoebae’. (**A**) Trophozoites as seen in hanging drop preparations (scale bar = 20 μm). (**B**) Fine structure of JAMX8 and its bacterial endosymbiont inhabiting the perinuclear space. (i) Section of an amoeba trophozoite: vesicular nucleus (n), phagosomes (p), mitochondria (m), bacterial endosymbionts associated with the nuclear envelope (arrowheads) (scale bar = 5 μm). (ii) Amorphous and tenuous cell coat (scale bar = 200 nm). (iii) Mitochondria with tubular cristae (scale bar = 1 μm). (iv) The Golgi complex organized as dictyosome (scale bar = 1 μm). (v,vi) Bacterial endosymbionts located within the perinuclear space, between inner and outer nuclear membrane. (v) Nucleus in detail with numerous endosymbiotic bacteria in transverse section (scale bar = 2 μm). Arrowheads indicate outer nuclear membrane. (vi) Longitudinal section through a rod-shaped bacterial endosymbiont, nucleus (n) (scale bar = 1 μm). (**C**) Fluorescence *in situ* hybridization image showing the co-localization of ‘*Candidatus* Nucleophilum amoebae’ (*Nucleophilum*-specific probe JAMX8_197, pink) with its host nucleus (DAPI, blue); food bacteria (general bacterial probe EUB338-mix, green) enclosed in the amoeba cytoplasm (probe EUK516, grey); scale bar indicates 10 μm.

The second isolate, strain JAMX8, showed flattened trophozoites with variable shape with an average length of 19.9 μm (S.D. 3.9, n = 26), width of 16.3 μm (S.D. 3.5, n = 26) and length/width ratio 0.88–1.84 (in average 1.25) (Fig. 3A). A frontal irregular hyaline zone occupied about one third of the cell length and was clearly separated from the granuloplasm containing a large quantity of spherical granules. The hyaloplasm possessed typically one to three longitudinal ridges. The locomotive cells often produced short dactylopodia (usually not more than 5 μm in length) that could freely move horizontally or vertically. No cysts were observed during subculturing. Floating forms consisted of a spherical central body with an average size of 4.8 μm in diameter (S.D. 0.7, n = 20) and thin radiating pseudopodia not longer than 10 μm (6.3 μm in average). A single, vesicular nucleus was located near the border of the granuloplasm (Fig. 3A,Bi,v). The cell surface was covered with a thin and amorphous cell coating (Fig. 3Bii). The cytoplasm contained numerous phagosomes (Fig. 3Bi), rounded or ovoid mitochondria (Fig. 3Bi,iii) with tubular cristae (Fig. 3Biii) and a Golgi complex organized as dictyosome (Fig. 3Biv). Comparison of the partial 18S rRNA sequence (2081 nt) of strain JAMX8 with known sequences revealed the absence of highly similar sequences in the NCBI nr/nt database. Taxa with moderate sequence similarity (<88%) were scattered among various amoebozoan lineages. In our phylogenetic analyses the JAMX8 strain represented a deeply branching novel lineage in the Amoebozoa with no clear affiliation to described taxa (Figs 2, S1).

Electron microscopy and staining with the DNA dye DAPI readily revealed the presence of bacterial endosymbionts in both amoeba strains (Figs 1 and 3).

### Bacterial endosymbionts in the amoeba cytoplasm and perinuclear space

In addition to ingested bacteria in food vacuoles (Fig. 1Bi), amoeba strain A1 harbored morphologically different rod-shaped bacteria with a diameter of about 0.44 μm and a maximum length of 1.2 μm (Fig. 1Bi,iv–vi). These bacteria were predominantly located enclosed in vacuoles (arrowheads in Fig. 1Biv,v), in which dividing cells were observed (Fig. 1Bv). The bacterial endosymbionts appeared to be few in numbers and scattered throughout the cytoplasm. However, nearly 100% of all amoeba trophozoites were infected.

Ultrastructural analysis of amoeba strain JAMX8 revealed bacterial symbionts at a conspicuous location within the cells (Fig. 3Bi,v,vi); rod-shaped bacteria of about 0.41 μm in diameter and a maximum length of 1.7 μm were found enclosed in the perinuclear space, between the inner and the outer nuclear membrane. Bacteria were never observed within the nucleoplasm. Nearly 100% of amoeba cells were infected. Both amoeba strains showed no apparent signs of symbiont-induced stress or lysis; the symbiotic associations could be stably maintained non-axenically in the lab.

### Novel gammaproteobacteria related to the *Coxiellaceae*

Sequencing of the 16S rRNA gene revealed that the endosymbiont of amoeba strain A1 showed highest 16S rRNA sequence similarity to *Legionella longbeachae* (85%) in the NCBI RefSeq database, which contains only sequence information of well described organisms^34^. The bacterial symbiont was tentatively named “*Candidatus* Occultobacter vannellae A1” (referring to the hidden location of these bacteria inside their *Vannella* sp. host and their small cell size; hereafter: *Occultobacter*). The endosymbiont of amoeba strain JAMX8 was most similar to *Coxiella burnettii* (88% 16S rRNA sequence similarity), and is provisionally referred to as “*Candidatus* Nucleophilum amoebae JAMX8” (referring to the association of these bacteria with the amoeba nucleus; hereafter: *Nucleophilum*). The rRNA sequences of both endosymbionts had a similarity of 85% with each other.

**Figure 4:**
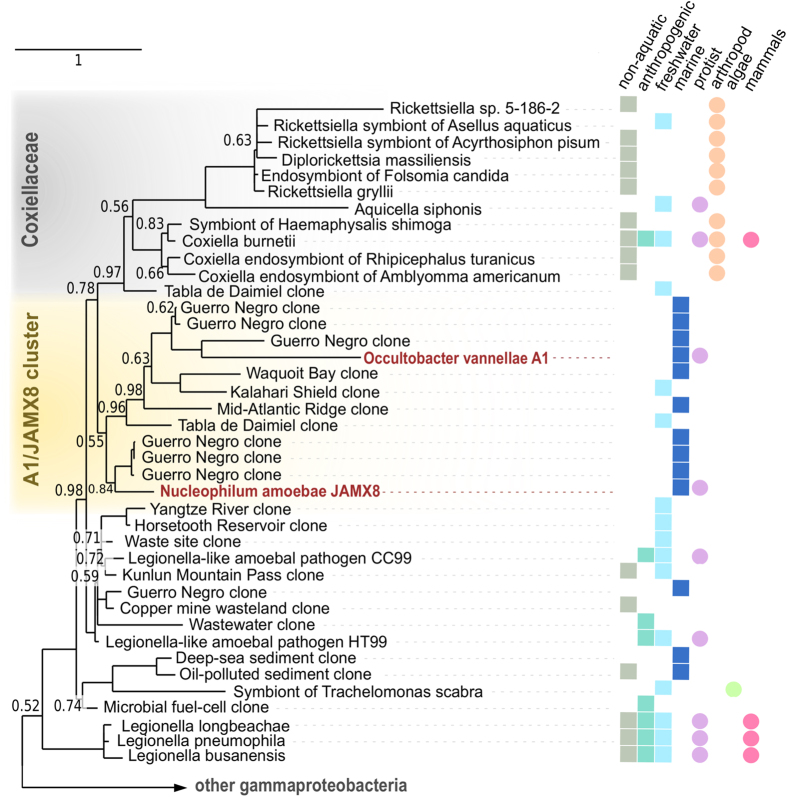
Phylogenetic relationship of ‘*Candidatus* Occultobacter vannellae’ and ‘*Candidatus* Nucleophilum amoebae’ with the *Gammaproteobacteria*. The phylogenetic tree (PhyloBayes, CAT + GTR) is based on the 16S rRNA sequences, Bayesian posterior probabilities are indicated at the nodes (only values < 0.99 are shown). Colored squares indicate the environmental origin of the respective sequence; colored circles indicate host association. A detailed version of this tree including accession numbers is available as supplementary Figure S2.

For phylogenetic analysis we first calculated trees with the CAT + GTR and the GTR models in PhyloBayes^33^. We then used posterior predictive tests to compare the fit of the models to the data (observed diversity: 2.866), indicating that CAT + GTR (posterior predictive diversity: 2.859 +/− 0.027, p-value: 0.57) was superior to the GTR (posterior predictive diversity: 3.138 +/− 0.027, p-value: 0). We therefore used the CAT + GTR model to assess the phylogenetic position of the two endosymbionts, demonstrating that both represent deeply branching lineages in the *Gammaproteobacteria* (Figs 4, S2). In our analysis they grouped with several marine and freshwater clones, together forming a sister clade to the *Coxiellaceae* (Bayesian posterior probability = 0.78). *Occultobacter* and *Nucleophilum* were also moderately related to a clade comprising the two unclassified amoeba-associated bacteria CC99 and HT99 (Bayesian posterior probability = 0.98)^35^. We searched published 16S rRNA amplicon and metagenomic sequence datasets using an approach described recently^63^, but did not find significant numbers of similar sequences to *Occultobacter* or *Nucleophilum* at a 97% similarity threshold.

In order to demonstrate the intracellular location of the bacterial symbionts FISH experiments were performed by combining symbiont-specific probes with a universal bacterial probe mix^20^. The positive hybridization reaction with both probes respectively confirmed the location of *Occultobacter* in the cytoplasm of its *Vannella* sp. A1 host (Fig. 1C) and the association of *Nucleophilum* with the nucleus of its JAMX8 amoeba host (Fig. 3C).

## Discussion

Here we report on the recovery of two novel stenohaline amoeba from marine samples. Based on light microscopy, amoeba strain A1 was readily identified as a member of the ubiquitous family Vannellidae whose members are also frequently found in marine environments^36^. Nuclear structure (laterally located nucleolus/nucleoli) and trophozoite size allow an assignment of this strain to *Vannella plurinucleolus* (Fig. 1). However, the shape of its cell surface is in conflict with the diagnostic features of *V. plurinucleolus*. Yet, in our phylogenetic analyses strain A1 clustered together with *V. plurinucleolus* strain 50745 (Fig. 2). As the taxonomy of *V. plurinucleolus* strain 50745 is under debate^37^, we decided to leave strain A1 undetermined at the species level. Morphological and molecular characterization of additional *Vannella* strains will be required to resolve species identification.

By light microscopy, trophozoites of strain JAMX8 showed a combination of morphological features typical of the genera *Mayorella* and *Korotnevella*^38^. However, neither essential diagnostic features, like a surface cuticle or surface microscales, nor any other distinct characteristics were found (Fig. 3). We were thus not able to assign strain JAMX8 to any described gymnamoeba species or genus based on morphological criteria. Furthermore, the placement of JAMX8 within the Amoebozoa could not be unambiguously determined in our phylogenetic analyses (Fig. 2). The deeper nodes in our Amoebozoa tree are generally rather unstable, which is consistent with previous studies^39^,^40^, and the tree topology was dependent on taxon sampling and alignment trimming stringency. However, strain JAMX8 groups with low statistical support with *Vermistella antarctica* (Bayesian posterior probability 0.69, maximum likelihood bootstrap value 16%), and this sister taxa relationship was recovered repeatedly in different analyses. Taken together, the exact relationship of strain JAMX8 to known amoebae remains elusive. However, morphological and phylogenetic data suggest that JAMX8 is a representative of a new taxon within the Amoebozoa.

To our knowledge this is the first molecular identification and characterization of bacterial symbionts of marine amoebae. In the last two decades numerous reports described the discovery of obligate intracellular amoeba symbionts^8^,^10^. However, these studies were often biased towards the isolation of *Acanthamoeba*, *Naegleria* or *Vermamoeba* (former *Hartmannella*) strains, usually from anthropogenic or freshwater habitats or clinical samples^7^,^41^,^42^. This is not surprising as these amoebae are in the focus of medical and parasitological research, and standard isolation protocols are available^2^,^16^,^43^,^44^. Interestingly, bacterial symbionts previously found in these amoebae were frequently very similar to each other; although isolated from geographically distant places, the endosymbionts belonged to symbiont clades either in the *Alpha*- or *Betaproteobacteria*, the *Bacteroidetes*, or the *Chlamydiae*^10^,^11^,^45^,^46^,^47^,^48^. In addition, *Gammaproteobacteria*, such as *Coxiella, Francisella, Legionella*, and *Legionella*-like amoebal pathogens, may also be associated with amoebae^7^,^48^,^49^,^50^, but these are mostly facultative associations. These bacteria show a parasitic life style, and many also infect higher eukaryotes^7^. Our study for the first time reports on *Gammaproteobacteria* naturally living in a stable association with their amoeba hosts, i.e. host and symbiont can together be maintained in culture over extended periods of time without apparent signs of host cell lysis and 100% of amoebae being infected.

The two gammaproteobacterial symbionts *Occultobacter* and *Nucleophilum* represent novel phylogenetically deeply branching lineages, with only low 16S rRNA sequence similarity to known bacteria (85% and 88%, respectively; Fig. 4). In addition, no close relatives (>97% 16S rRNA sequence similarity) could be retrieved in any of the numerous 16S rRNA amplicon studies targeting biodiversity of marine environments, suggesting that both endosymbionts are rather rare and/or their hosts have not been captured during sampling. Our phylogenetic analysis showed that the exact position of the two endosymbionts in the *Gammaproteobacteria* is difficult to resolve. *Occultobacter* and *Nucleophilum* group together in a well-supported monophyletic clade with the *Coxiellaceae* and a cluster comprising the *legionella*-like amoebal pathogens HT99 and CC99 (Fig. 4). The topology within this clade is, however, not very robust. This is in agreement with previous observations, highlighting the challenge of resolving the phylogeny of the major *Gammaproteobacteria* groups^51^.

The closest relatives of *Occultobacter* and *Nucleophilum* are other symbionts and pathogens of eukaryotes. The *Coxiellaceae* mainly include bacteria infecting arthropods, which occasionally also invade mammalian or protozoan hosts^49^,^52^,^53^. The bacteria referred to as HT99 and CC99 were associated with amoebae found in a hot tub and a cooling tower, respectively^35^. Worth noting, while the *Coxiellaceae*, CC99, HT99 and related taxa mainly originate from freshwater, anthropogenic and non-marine habitats, many of the closest relatives of *Occultobacter* and *Nucleophilum* were detected in marine environments (Fig. 4).

The two symbionts described here colonize fundamentally different intracellular niches. Whereas, similar to many known intracellular bacteria, *Occultobacter* establishes replication inside host-derived vacuoles and is also occasionally found as single cell inside the cytoplasm, *Nucleophilum* is associated with its host cell’s nucleus (Figs 1 and 3). The latter is a very unusual life style^54^,^55^, but there are few reports on bacteria located in the nuclear compartment of other amoebae, namely the chlamydial symbiont of *Naegleria* ‘Pn’^56^,^57^, the two gammaproteobacteria HT99 and CC99^35^, and the alphaproteobacterium *Nucleicultrix amoebiphila*^58^. Bacteria capable to invade the nucleus possibly benefit from a nutrient-rich environment, protection from cytoplasmic defense mechanisms and a direct path to vertical transmission during host cell replication^55^. However, the pattern of how bacteria settle in this compartment shows striking differences; while *Nucleicultrix* is spread out in the nucleoplasm, Pn is associated with the nucleolus, and *Nucleophilum* is located in the perinuclear space^56^,^57^,^58^. Embedded in between the inner and outer nuclear membrane, the bacteria thus do not have direct access to the nucleoplasm. The perinuclear space, which is continuous with the endoplasmic reticulum, serves as a calcium storage^59^ and has regulatory impact on processes in the nucleus, such as gene expression^60^. The exact physicochemical conditions of this compartment remains currently unknown^59^, however, in contrast to the nucleoplasm or the cytoplasm it likely contains less substrates to support bacterial growth. We therefore expect *Nucleophilum* to have evolved unconventional strategies to target its peculiar perinuclear niche and to satisfy its nutritional requirements. Previously, it has been shown and hypothesized that some intranuclear bacteria confer beneficial effects to their hosts, such as an increased survival under adverse environmental conditions or protection against co-infection by cytoplasmic bacteria^55^,^61^,^62^. However, at the moment we have no indication that the symbiosis between *Nucleophilum* and its amoeba host is a mutualistic association. Genome analysis in combination with functional approaches, such as transcriptomics and proteomics, will help to gain insights into the infection process, interaction mechanisms, possible benefits for host and symbiont, and the evolution of this unique lifestyle.

This is the first report on the concomitant isolation and characterization of marine amoebae and their bacterial endosymbionts. The low degree of relationship of the symbionts to known bacteria and the discovery of a symbiont thriving in the host perinuclear space, a niche not reported previously for intracellular microbes, indicates that marine habitats represent a rich pool of hidden symbiotic associations.

## Additional Information

**How to cite this article**: Schulz, F. *et al*. Marine amoebae with cytoplasmic and perinuclear symbionts deeply branching in the *Gammaproteobacteria*. *Sci. Rep*. **5**, 13381; doi: 10.1038/srep13381 (2015).

## Supplementary Material

Supplementary Information
