# Supplementary material for: Marine amoebae with cytoplasmic and perinuclear symbionts deeply branching in the Gammaproteobacteria
Source: Sci Rep. 2015 Aug 25;5:13381. doi: 10.1038/srep13381 (PMC4642509; doi:10.1038/srep13381)
Supplement: Supplementary Information [file srep13381-s1.pdf]

Marine amoebae with cytoplasmic and perinuclear symbionts deeply branching in the *Gammaproteobacteria*

Frederik Schulz[1†], Tomáš Tyml[2,3,4†], Ilaria Pizzetti[5], Iva Dyková[4], Stefano Fazi[5], Martin Kostka[2,3], Matthias Horn[1*]

[1]Department of Microbiology and Ecosystem Sience, University of Vienna, Althanstraße 14, A-1090 Vienna, Austria

[2]Faculty of Science, University of South Bohemia, Branišovská 31, 370 05 České Budějovice, Czech Republic

[3]Institute of Parasitology, Biology Centre CAS, Branišovská 31, 370 05 České Budějovice, Czech Republic

[4]Department of Botany and Zoology, Faculty of Science, Masaryk University, 61137 Brno, Czech Republic

[5]Water Research Institute, National Research Council of Italy (IRSA-CNR), Via Salaria Km 29,300–, 00015 Monterotondo - Roma, Italy

†these authors contributed equally to this work

*Corresponding author: Department of Microbiology and Ecosystem Science, University of Vienna, Althanstrasse 14, A-1090 Vienna, Austria, Phone: +43 1 4277 76608, horn@microbial-ecology.net

Supplementary Material

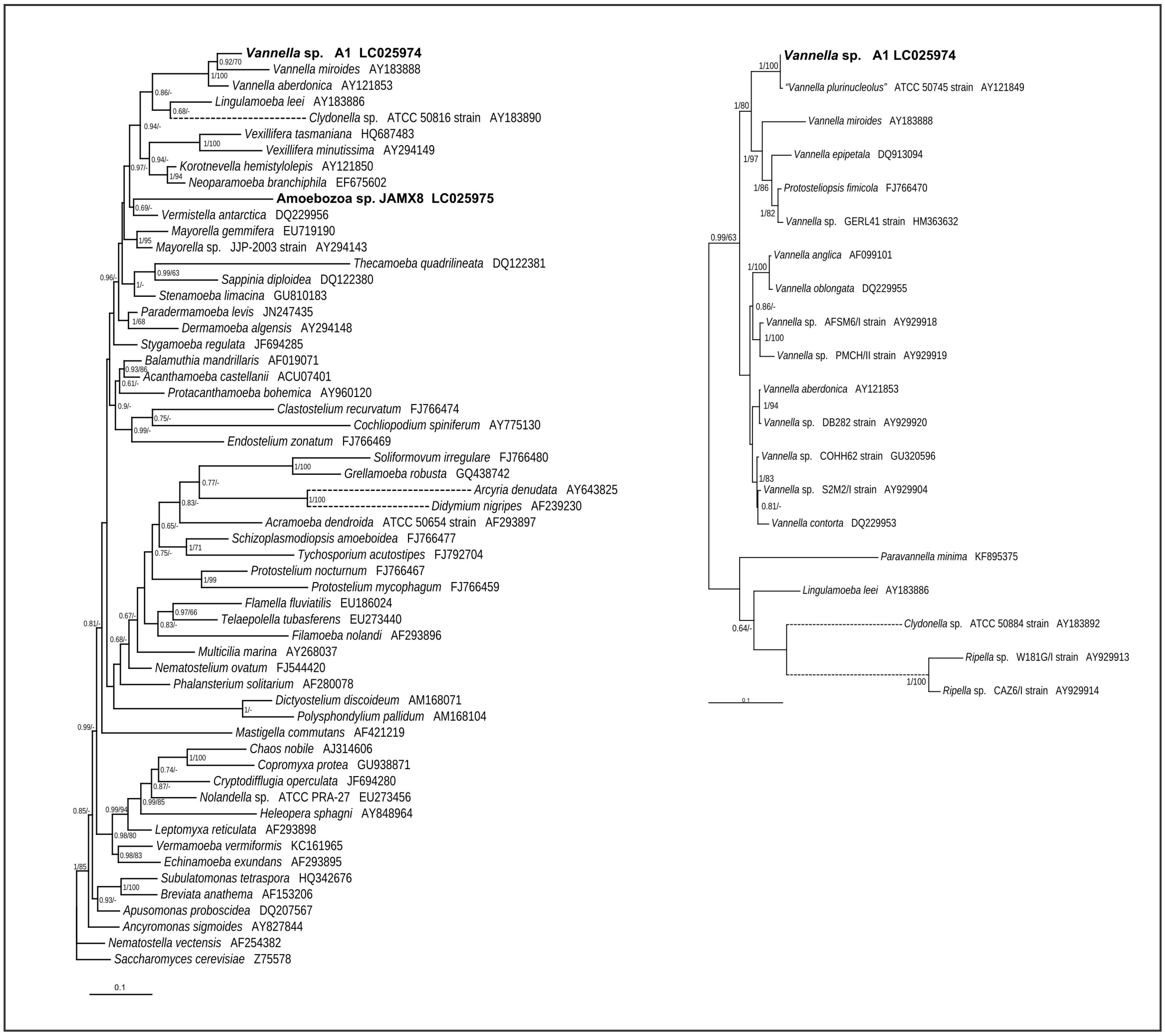

Figure S1. Phylogenetic relationships of *Vannella* sp. A1 and amoeba isolate JAMX8 within the Amoebozoa. Phylogenetic 18S rRNA-based trees of the Amoebozoa (left panel) and Vannellidae (right panel) constructed using the Bayesian inference method. Bayesian posterior probabilities 8 (> 0.6) and RaxML bootstrap support values (> 60%) are indicated at the nodes; the dashed line indicates a branch shortened by 50% to enhance clarity.

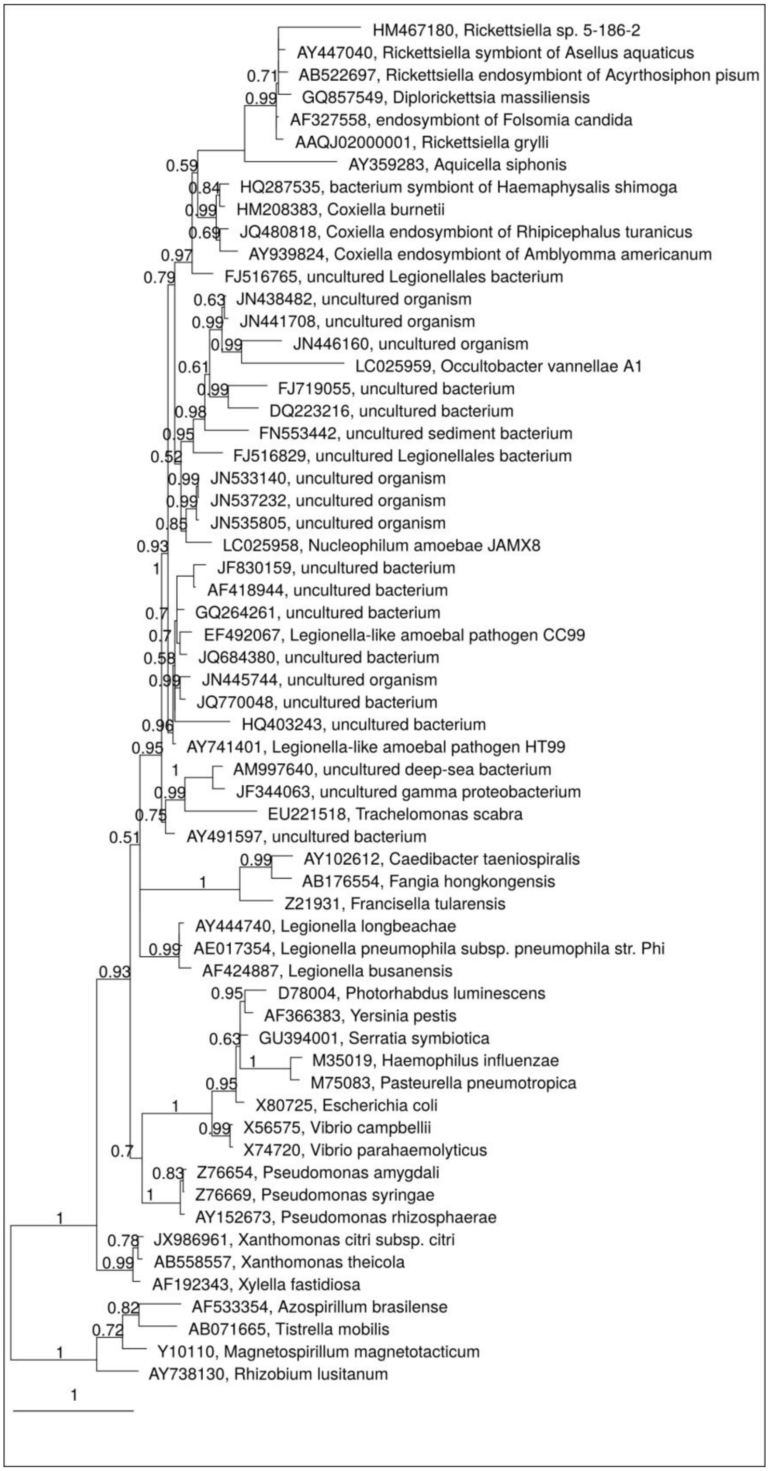

Figure S2. Phylogenetic relationship of '*Candidatus* Occultobacter vannellae' and '*Candidatus* Nucleophilum amoebae' with the *Gammaproteobacteria*. The phylogenetic tree (Phylobayes, CAT+GTR) is based on the 16S rRNA sequences, Bayesian posterior probabilities are indicated at the nodes.
